# Supplementary material for: Dietary Supplementation of Foxtail Millet Ameliorates Colitis-Associated Colorectal Cancer in Mice via Activation of Gut Receptors and Suppression of the STAT3 Pathway
Source: Nutrients. 2020 Aug 7;12(8):2367. doi: 10.3390/nu12082367 (PMC7468867; doi:10.3390/nu12082367)
Supplement: Supplementary file 1 [file nutrients-12-02367-s001.pdf]

Table S1. Operating parameters in MRM-mode.

Compound	Molecular formula	M (g/mol)	M + X+ (m/z)	Fragments (m/z)	DP (V)	EP (V)	CE (V)
3-methylindole (3ML)	C_9H_9N	131.17	M + H+	Q1 132.20 → 63.00<br>Q2 132.20 → 117.00	30<br>23	9<br>12	21<br>27
5-hydroxytryptamine (5HT)	$C_{10}H_{12}N_2O$	176.21	M + H+	Q1 177.20 → 160.20<br>Q2 177.20 → 132.00	16<br>16	6<br>8	20<br>30
5-Hydroxytryptophan (5-HTP)	$C_{11}H_{12}N_2O_3$	220.22	M + H+	Q1 221.20 → 204.10<br>Q2 221.20 → 162.20<br>Q2 177.20 → 132.00	22<br>21	4<br>4	17<br>24
3-Indoleacrylic acid (IA)	$C_{11}H_9NO_2$	187.19	M + H+	Q1 188.20 → 146.10<br>Q2 188.20 → 160.10<br>Q3 188.20 → 170.20	35<br>36<br>16	12<br>6<br>9	21<br>18<br>18
indole acetate (IAA)	$C_{10}H_9NO_2$	175.18	M + H+	Q1 176.10 → 130.00<br>Q2 176.10 → 103.10<br>Q3 176.10 → 77.10	30<br>30<br>30	10<br>10<br>10	35<br>35<br>35
Indole-3-Acetamide (IAM)	$C_{10}H_{10}N_2O$	174.20	M + H+	Q1 175.20 → 130.20<br>Q2 175.20 → 105.00	29<br>30	5<br>5	18<br>12
indole-3-carboxaldehyde (Icld)	C_9H_7NO	145.16	M + H+	Q1 146.10 → 118.00<br>Q2 146.10 → 118.00	24<br>35	10<br>6	21<br>15
indole lactate (ILA)	$C_{11}H_{11}NO_3$	205.21	M + H+	Q1 206.20 → 170.10<br>Q2 206.20 → 118.30	33<br>33	10<br>10	30<br>30
Indole	C_8H_7N	117.15	M + H+	Q1 118.00 → 91.00<br>Q2 118.00 → 63.00	9<br>9	10<br>10	30<br>24

3-Indolepropionic acid (IPA)	$C_{11}H_{11}NO_2$	189.21	M + H$^+$	Q1 190.20 → 130.30<br>Q2 190.20 → 172.30	29<br>25	12<br>8	26<br>21
Kynurenine (Kyn)	$C_{10}H_{12}N_2O_3$	208.21	M + H$^+$	Q1 209.00 → 192.00<br>Q2 209.00 → 146.00<br>Q3 209.00 → 94.00	11<br>29<br>25	9<br>8<br>8	12<br>27<br>21
Melatonin (ST)	$C_{13}H_{16}N_2O_2$	232.28	M + H$^+$	Q1 233.10 → 174.00<br>Q2 233.10 → 130.00<br>Q3 233.10 → 159.00	60<br>60<br>60	12<br>12<br>12	20<br>47<br>36
Typtophan (Trp)	$C_{11}H_{12}N_2O_2$	204.22	M + H$^+$	Q1 205.00 → 118.00<br>Q2 205.00 → 115.10	120<br>130	10<br>10	24<br>46

Table S2. Sequence of the primers used in this study [sequence 5′-3′].

	Forward	Reverse
β-actin	ACAGCAGTTGGTTGGAGCAA	ACGCGACCATCCTCCTCTTA
18S	AAGGGCTGCTTCCAA ACC TTT GAC	TGCCTGAAGCTCTTGTTGATGTGC
IL-1β	AAGGGCTGCTTCCAAACCTTTGAC	TGCCTGAAGCTCTTGTTGATGTGC
IL-6	CTCTGGCGGAGCTATTGAGA	AAGTCTCCTGCGTGGAGAAA
IL-10	CAGGACTTTAAGGGTTACTTG	ATTTTCACAGGGGAGAAATC
IL-17	ACGTTTCTCAGCAAACTTAC	CCCCTTTACACCTTCTTTTC
IL-22	GAGGCCAGCCTTGCAGATAA	CAGGAGCTGAGCTGATTGCT
TNF-α	CGTGGAACTGGCAGAAGAGG	CAGGAATGAGAAGAGGCTGAGAC
IFN-γ	GCTCTGAGACAATGAACGCTACAC	TTCTTCCACATCTATGCCACTTGAG
COX-2	CCCATTAGCAGCCAGTTGTC	CAGGATGCAGTGCTGAGTTC
iNOS	GGGCTGACCTGTTTCCTACT	GGAGGTTGAGACCCAATGGA
ZO-1	GGGGCCTACACTGATCAAGA	TGGAGATGAGGCTTCTGCTT
Occludin	ACGGACCCTGACCACTATGA	TCAGCAGCAGCCATGTACTC
AHR	GAGCTTCTTTGATGGCGCTG	CCTTGTGCAGAGTCTGGGTTT
GPR41	TCCAGCCTGGCTTTCCAATA	GCCTGCAGGAGACATTTCAG
GPR43	CGACTAGAGATGGCTGTGGT	AGAAGATGAGCAGTGTGGCT
FOXP3	AATAGTTCCTTCCCAGAGTTC	GGTAGATTTCATTGAGTGTCC
GPR109a	AGTGGAAACTTCTCAGCCTTCCAA	TCCCCTCCAGTTTCGTGGA
VEGFa	CTGCCTGGAAGAATCGGGAG	GTACCCAGGAGGTGGGGTAA

Table S3. Difference of the values of PC1 between data from different cages within the same group.

	P **value for PC1 (43.9%), Cage 1 vs Cage 2**
NM	0.403
AOM/DSS	0.772
AOM/DSS+millet	0.860
AOM/DSS+rice	0.700

Statistical difference was analyzed by Wilcoxon rank-sum test. n=10, 6, 7, and 4 for the NM, AOM/DSS, AOM/DSS+millet, and AOM/DSS+rice groups, respectively.

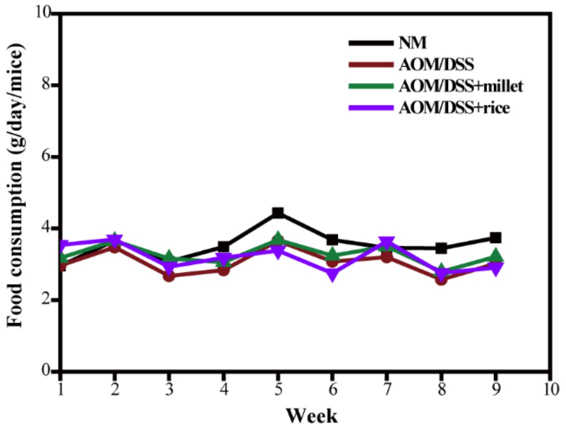

Figure S1. Daily food consumption per mouse in each group. The average food consumption was calculated based on the total food intake of the mice within a single cage.

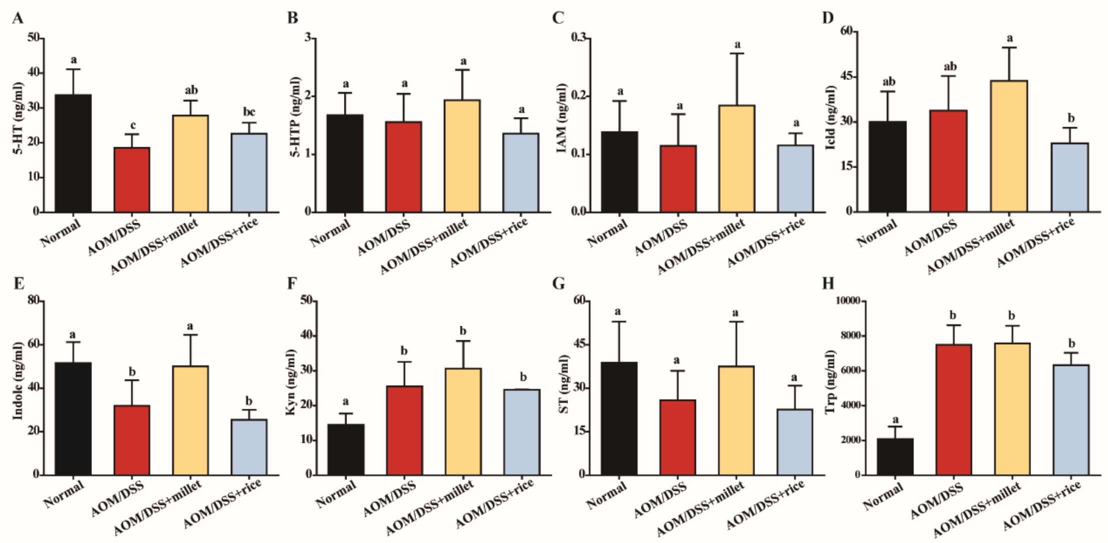

Figure S2. The concentrations of other tryptophan metabolites in feces. (A)5-HT, (B)5-HTP, (C)IAM, (D)Icld, (E)Indole, (F)Kyn, (G)ST and (H)tryptophan. Data are shown as the mean ± SD. Means with different letters are significantly different ($p < 0.05$).

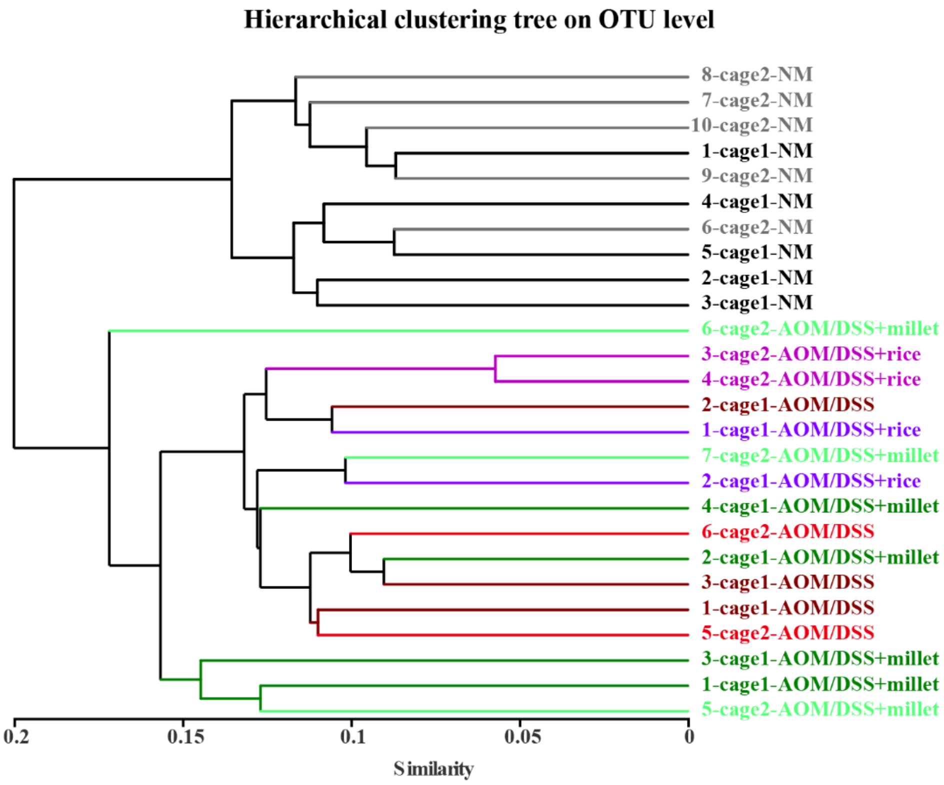

Figure S3. Cluster analysis of gut microbiota at OTU level with Unweighted-Unifrac distance.
